# Properties and prospects of adjuvants in influenza vaccination - messy precipitates or blessed opportunities?

**DOI:** 10.1186/2052-8426-1-2

**Published:** 2013-11-06

**Authors:** Babak Jalilian, Stig Hill Christiansen, Halldór Bjarki Einarsson, Mehdi Rasoli Pirozyan, Eskild Petersen, Thomas Vorup-Jensen

**Affiliations:** Biophysical Immunology Laboratory, Department of Biomedicine, Aarhus University, DK-8000 Aarhus, Denmark; Department of Clinical Medicine, Aarhus University, Aarhus, Denmark; Inflammation and Infection Research Centre, School of Medical Sciences, University of New South Wales, Sydney, Australia; Department of Infectious Medicine (Q), Aarhus University Hospital, Aarhus, Denmark

**Keywords:** Adjuvants, Influenza vaccination, Particle size

## Abstract

Influenza is a major challenge to healthcare systems world-wide. While prophylactic vaccination is largely efficient, long-lasting immunity has not been achieved in immunized populations, at least in part due to the challenges arising from the antigen variation between strains of influenza A virus as a consequence of genetic drift and shift. From progress in our understanding of the immune system, the mode-of-action of vaccines can be divided into the stimulation of the adaptive system through inclusion of appropriate vaccine antigens and of the innate immune system by the addition of adjuvant to the vaccine formulation. A shared property of many vaccine adjuvants is found in their nature of water-insoluble precipitates, for instance the particulate material made from aluminum salts. Previously, it was thought that embedding of vaccine antigens in these materials provided a “depot” of antigens enabling a long exposure of the immune system to the antigen. However, more recent work points to a role of particulate adjuvants in stimulating cellular parts of the innate immune system. Here, we briefly outline the infectious medicine and immune biology of influenza virus infection and procedures to provide sufficient and stably available amounts of vaccine antigen. This is followed by presentation of the many roles of adjuvants, which involve humoral factors of innate immunity, notably complement. In a perspective of the ultrastructural properties of these humoral factors, it becomes possible to rationalize why these insoluble precipitates or emulsions are such a provocation of the immune system. We propose that the biophysics of particulate material may hold opportunities that could aid the development of more efficient influenza vaccines.

## Introduction

Seasonal influenza is one of the most common infections in humans. In general, symptoms are mild. However, elderly people and patients with a compromised immune response, or an otherwise impaired health may ultimately succumb to severe complications of the infection. For this reason, there is an important and continuous need for vaccine development, and production. Influenza vaccines only provide strain specific protection. Because of antigenic drift, vaccines are tailored to the present circulating strains each year. A change of vaccines is also needed when antigenic shift occurs, and a new pandemic arise. With the challenges in production and distribution in mind, a pan-protective influenza vaccine providing long-term protection would be a huge step forward.

In this paper, we argue that one strategy to achieve this goal is through the use of better adjuvants, in particular these inducing an immune response mimicking the natural infection.

Several decades of research have unravelled the mechanisms of the immune system, which are important in protecting against influenza virus infection. It is now clear these mechanisms prominently involve contributions from both the innate and adaptive immune system. In clinicallyused and experimental vaccines, there is considerable uniformity in the choice of antigenic components in influenza vaccines, and hence how to guide the adaptive immune response in fighting the virus. Some vaccines include adjuvants, which largely act by stimulating the innate immune system, thereby priming a stronger response by the adaptive immune system. Interestingly, the choices of adjuvants are chemically diverse, but often with a particulate nature, prompting C.A. Janeway (1943–2003) famously to refer to these as “messy precipitates” [1].

Here, we discuss current trends in the development of adjuvants for use with influenza vaccines. In “Introduction” section, a brief outline is presented on the clinical manifestations and viral biology of influenza virus together with mechanisms of the immune system limiting the infection. In “Adaptive immunity and formulation of antigenic components in influenza vaccines” section, some of the vaccine antigens used, and their route of administration are compared. Finally, “Innate immunity and immunogenic and physicochemical properties of adjuvants” section reviews the pharmacological mode-of-action of clinicallyused, and experimental adjuvants in influenza vaccines by elaborating on the potential for adjuvant particle-size-related immunomodulatory stimuli.

### Clinical manifestations of influenza virus infections

Droplet transmission and physical contact with virus-contaminated surfaces seems to be the primary means of influenza virus dissemination prior to inoculation [2]. The incubation time is approximately 1 to 3 days [3]. Cardinal manifestations of disease are malaise and headache during the prodromal period. This is followed by a sore throat, due to laryngotracheobronchitis and hyperaemic, or erythematous oral and pharyngeal mucous membranes associated with catarrhalia, rapid increase in body temperature, non-productive cough, dedolation, chills, and myalgia. The severity of the symptoms depends on the degree of immunity raised by past infections. Pre-existing conditions disposing a more severe disease include pregnancy where seasonal influenza provokes a 3–4 fold increased risk of cardiopulmonary illness, notably in the third trimester [4]. Chronic lung diseases or affected respiratory function due to respiratory muscle atrophy, cardiovascular diseases, diabetes, chronic liver and kidney diseases [5], acquired immunodeficiency [6], or obesity [7] also predispose to an aggravated course of the infection. Children infected with influenza usually present the same symptomatology as in adults, however, frequently with higher body core temperature, croup, otitis media, bronchiolitis, abdominal pain, and vomiting. Furthermore, unlike in adults, influenza-associated mortality in children appears not to be associated with an underlying medical condition [8]. Persistent fever, *i.e.*, for longer than five days, may be a consequence of bacterial co-infections with *Streptococcus pneumonia*, *Haemophilus influenza* and *Staphylococcus aureus.* These pathogens cause sinuitis, otitis, bronchitis and/or pneumonitis. Co-infections with *Staphylococci* are fulminant and even lethal [9, 10]. In more general, the bacterial pathogens may cause septic shock as well as exacerbate pulmonary and cardiac diseases [11–13].

### Architecture and strain diversity of the influenza virus

Influenza viruses are the only members of the *Orthomyxoviridae* family. They are classified into three different *genera*, A, B and C, based on the expression of matrix (M_1_) protein, membrane (M_2_) protein and nucleoprotein (NP). *Orthomyxoviridae* viruses have several biological properties in common. However, they differ significantly in their host tropism [14]. Influenza viruses of *genera* B and C principally infect humans, although they occasionally have been isolated from seals and pigs [15, 16]. Influenza A viruses, on the other hand, propagate in several animal hosts, including humans, pigs, horses, minks, and in domestic and wild birds. Waterfowls are the primary reservoirs, in which the virus is hosted in the intestine. Several species act as mixing vessel between humans and birds, primarily the pig, which express receptors for both human and avian viruses in their upper respiratory tract epithelial cells. In general, influenza A viruses are nonpathogenic in birds and are classified as either low or high pathogenic avian influenza viruses (LPAI or HPAI, respectively), depending on the morbidity and mortality upon transmission to other species, including humans [17].

**Figure 1:**
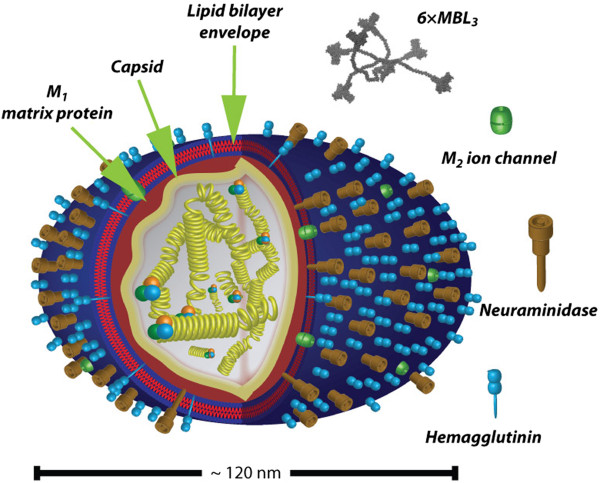
**Architecture of the influenza virion.** While the virus has often been depicted as spherical in microbiology textbooks [14], recent investigations suggest a far more pleiomorph appearance, only some particles being essentially spherical while others are tubular. The approximate length of the virion is 120 nm, possibly influenced by the organization of the RNA by the multiple ribonucleoprotein complexes [19] shown in the center of virion surrounded by the capsid and layer of matrix protein M_1_. The relative abundance of the HA and NA proteins are indicated based on reports by Mitnaul *et al.*[20] and Zhang *et al.*[21] (components not to scale). Based on data from the report by Gjelstrup *et al.*[22], a 6×MBL_3_ oligomer is drawn to scale relative to the size of the influenza virion.

Subtyping of influenza viruses is based on the antigenic properties of their surface hemagglutinin (HA) and neuraminidase (NA) glycoproteins. Currently, 17 different subtypes of HA have been identified, of which the most recent (H_17_) was identified in fruit bats [18]. Nine different subtypes of NA are known. The influenza virion (Figure 1) contains a linear, negative sense, single-stranded RNA with 7 (C *genus*) or 8 (A and B *genera*) segments, each expressing one or two of the influenza virus proteins. The segmented genome of the influenza virus facilitates genomic changes by genetic drift and/or genetic shift. Genetic drift refers to minor changes due to mutation in the genes encoding HA or NA, altering viral antigenicity. Such alterations are sufficient to permit infections in individuals with immunity to similar viruses, and drift explains the regular outbreaks of seasonal influenza. Genetic shifts are major changes as a result of reassortment of genome segments from different human and/or animal strains producing anything from slightly to completely different influenza virus genomes. In this situation little pre-existing immunity to the virus can be found in human populations, potentially leading to severe pandemics. The latter process is restricted to virus of the A *genus*[14].

### Viral infectivity, treatment, and immunity to influenza

The requirements for influenza A virus infectivity are well studied. These viruses depend on sialic acid expression on the host tissue, which permits the attachment of the viral HA proteins. Human influenza virus mainly infects non-ciliated cells in the respiratory tract, while the avian virus infects ciliated cells [23], thereby reflecting the carbohydrate selectivity of HA. The avian viruses prefer attachment to galactose residues forming an α-(2,3)-glycosidic linkage to the sialic acid, while human viruses preferentially bind to galactose residues forming an α-(2,6)-linkage [24]. Pigs have both types of sialylations in their upper respiratory tract, while humans express α-(2,6)-galactose linked sialic acid in the upper respiratory tract and α(2,3)-galactose linked sialic acid in the lower respiratory tract [25]. NA activity is essential both to ensure the release of virus from infected cells, and to facilitate the development of the infection by providing access to the deeper layers of tissue, *i.e.* below the sialylated lung mucus [26].

Following infection with the influenza virus, inflammatory cells of the innate immune system accumulate in the mucosal membrane. The cellular response to infection involves hyperemia of the epithelium and necrosis in bronchiolar epithelial cells [27]. At least in part, this response is a consequence of the recruitment of neutrophil granulocytes to the site of inflammation and viral activation of epithelial cells [28]. The recruitment of granulocytes depends on the proteolytic cleavage of humoral factors of the innate immune system, particularly the group of plasma proteins constituting the complement system. Small fragments of these proteins, notably C5a, permeate the endothelium of adjoining blood vessels in the zone of inflammation and create a chemokine gradient guiding the granulocytes [29]. It is a classic finding that mannan-binding lectin (MBL, also known as mannose-binding lectin or mannan or mannose-binding protein, MBP), a complement-activating plasma protein, is significant in the protection against influenza infection in ferrets [30, 31]. Evidence also suggests that MBL plays a similar role in infections in humans [32]. MBL recognizes a particular topological pattern of glycans on HA, thereby triggering complement activation through the lectin pathway [31, 33, 34]. Opsonization through the deposition of proteolytic fragments of complement component C3 allows neutrophil granulocytes and other myeloid cells to the viral particles through complement receptor (CR)3 (also known as integrin α_M_β_2_, Mac-1, or CD11b/CD18) and CR4 (also known as integrin α_X_β_2_, p150,95 or CD11c/CD18) [35]. Other pattern recognition molecules, such as Toll-like receptors (TLRs), recognize the single-stranded RNA transported into infected cells by the influenza virus [36], which alerts cellular parts of the immune system.

In addition to mediating direct clearance of influenza virus, the innate immune system is also important in priming a response by mechanisms of adaptive immunity. Dendritic cells (DC) transport viral antigens into the lymph nodes, where naïve T cells are converted into influenza antigen-reactive CD8+ cytotoxic T lymphocytes (CTL) or CD4+ T helper (Th) cells. T cell responses are critical for protection against influenza infection, as was established in a classic study on human volunteers infected with an unattenuated strain of influenza virus [37]. Perhaps surprisingly, a recent analysis showed that CD4+ T cells with an interferon-γ-producing Th1 phenotype, and only to a lesser extent CTLs, are particularly important in protection in humans [38]. Influenza vaccines are evaluated by their ability to react with antibodies [39]. While there is evidence that antibody titers to vaccine antigens correlate with vaccine efficacy [39], the recent identification of CD4+ Th1 cells as important in protection against influenza infection [38] could raise a question if antibody titers are a sufficiently comprehensive measure of vaccine-induced immunity to infection. At the very least, vaccine strategies permitting formation of such influenza virus antigen-reactive T cells should be carefully considered as discussed in “Adaptive immunity and formulation of antigenic components in influenza vaccines” section.

Mature naïve T cells are maturated inside the thymus. Thymic function declines, however, with age, due to a gradual replacement of thymopoietic tissue with adipose tissue [40–42]. Thus, it is not surprising that impaired formation of antigen-specific antibodies in the elderly was reported for influenza vaccination [42, 43]. Although the total size of the peripheral lymphocyte pool is stable throughout life, the composition of both CD4+ and CD8+ T cells with regard to naïve and memory subpopulations changes with age [42, 44]. A modest decline of naïve CD4+ lymphocytes is observed until the ages of 65–75, due to homeostatic control mechanisms, but subsequently the naïve CD4+ T cell reserve collapses [42, 45]. Consistent with this, changes within the CD4^+^ naïve and memory subsets have been shown to impair long-term CD4^+^ T cell responses to influenza and hepatitis B vaccination [42, 46, 47]. These observations are particularly important in view of the essential role of these cells in protecting against the infection [38, 42].

At the molecular level one of the most profound biological indicators of ageing in the human immune system is the progressive loss of expression of the co-stimulatory molecule CD28 on T cells that have undergone repeated antigen stimulations [42, 48–50]. However, other mechanisms may be affected as well. In vaccinees younger than 35 years, expression of the adhesion molecule CD62L was observed to correlate with the ability to raise immunity to the hepatitis B virus following vaccination with hepatitis B surface antigen. The association between expression of the adhesion molecule CD62L (L-selectin) on CD4^+^ naïve and central memory T cells and the formation of antigen-specific antibodies was not found for donors older than 55 years [47]. Age-related alterations in the vaccine responsiveness may consequently affect vaccinees far younger than previously thought [42, 47]. Since the function of CD62L pertains to general functions of T cells, it is possible that the observations by Rosenberg *et al.*[42, 47] pertain to a wider number of vaccinations than those protecting against hepatitis B virus, and this also includes influenza vaccinations.

## Adaptive immunity and formulation of antigenic components in influenza vaccines

Advancements in understanding the etiology of infectious diseases and especially how antigen responses are formed through the adaptive immune system have provided means for novel ways of vaccine production and administration into the human body. The success of these accomplishments includes elimination of small pox as a human infection and efficient vaccines against other viruses providing life-long protection. Indeed, the concept of vaccination is no longer limited to prevention of infectious diseases, but is also used in modulating immune responses in autoimmune diseases [51]. With the live-attenuated small pox vaccine as an important example, vaccines broadly stimulating the immune system in a manner resembling the natural infection are far superior to most sub-component based, or otherwise engineered vaccines. By contrast, the prophylactic influenza vaccination has not achieved to induce long-lasting immunity in immunized populations [52]. To overcome this problem, the strategy has focused on challenging the adaptive immune response with influenza virus antigens tailored to the predicted upcoming seasonal or pandemic influenza virus, although the need for adequate supplies of vaccines and frequent vaccination hinder efforts to obtain sufficient protection. As an indication of load of vaccines supplied for single season, the United States of America federal Food and Drug Administration-recommended influenza vaccinations for the season 2013–2014 are listed in Table 1. It is tempting to suggest that inability to achieve long-lasting protection is in consequence of the wide use of inactivated or sub-component based formulations. However, the live-attenuated influenza vaccine FluMist (Table 1) also requires annual administration, suggesting that the biology of the virus is the real culprit in limiting vaccine efficiency. Below, we list major strategies for producing the antigenic component of influenza viruses.

**Table 1:** **Influenza virus vaccines for the United States of America 2013–2014 season** ^a^

Seasonal influenza vaccines	Type/abbrev.	# Antigens	Route of administration	Manufacturer	Age range	Adjuvant
Afluria	Inactivated/IIV3^b^	Trivalent	Intramuscular	CSL Limited	5 ≥	None
FluLaval	Inactivated/IIV3^b^	Trivalent	Intramuscular	GlaxoSmithKline	18 ≥	None
Fluarix	Inactivated/IIV3^b^	Trivalent	Intramuscular	GlaxoSmithKline	3 ≥	None
	Inactivated/IIV4^b^	Quadrivalent	Intramuscular			None
Flublok	Recombinant/RIV3^c^	Trivalent	Intramuscular	Protein Sciences	18-49	None
Flucelvax	Cell Culture/ccIIV3^b^	Trivalent	Intramuscular	Novartis	18 ≥	None
FluMist	Live Attenuated/LAIV4^d^	Quadrivalent	Intranasal	Medimmune	2-49	None
Fluvirin	Inactivated	Trivalent	Intramuscular	Novartis	4 ≥	None
Fluzone	Inactivated/IIV3^b^	Trivalent	Intramuscular	Sanofi Pasteur	6 mo ≥^e^	None
	Inactivated/IIV4^b^	Quadrivalent	Intramuscular			None

^a^U.S. Food and Drug Administration recommends that the trivalent-formulation influenza vaccines for the U.S. 2013–2014 influenza season contain the following: (1) A/California/7/2009 (H1N1)-like virus, (2) (H3N2) virus antigenically like the cell-propagated prototype virus A/Victoria/361/2011, and (3) B/Massachusetts/2/2012-like virus. For the quadrivalent-formulation influenza vaccines for the U.S. 2013–2014 influenza season contain the above three strains and the following additional B strain: (4) B/Brisbane/60/2008-like virus. ^b^IIV refers to inactivated vaccines (egg and cell-culture based). Includes trivalent (IIV3) and quadrivalent (IIV4). ^c^RIV refers to recombinant HA influenza vaccine. Trivalent (RIV3). ^d^LAIV refers to Live Attenuated Influenza Vaccine. Quadrivalent (LAIV4). ^e^Dose dependent. Informations were found on web sites: http://www.emergency.cdc.gov/coca/ppt/2013/08_13_13_Immunizations.pdf, http://www.fda.gov/biologicsbloodvaccines/guidancecomplianceregulatoryinformation/post-marketactivities/lotreleases/ucm343828.htm (accessed on 4 September 2013).

### Protein-based vaccines against influenza

Influenza vaccine antigens are commonly formulated either as whole inactivated virus or *in vitro*-expressed viral protein subcomponents [53].

Inactivated whole seasonal influenza vaccines are the most widely used, covering approximately 90% of human vaccine sales world-wide [54]. A new avian influenza vaccine is normally made by inoculation of the isolated virus into embryonated chicken eggs. The manufacturing process has been described in detail elsewhere [54]. Briefly, the allantoic fluids from influenza virus-inoculated embryonated eggs are collected in a fully automated process followed by a simple clarification step. The virus is recovered through multiple filtration and concentration steps and inactivated with chemicals such as formalin or β-propiolactone, typically in two steps [54]. The final influenza vaccine is trivalent with two strains of Influenza A virus (H3N2 and H1N1) and one strain of Influenza B virus to provide a suitable antigenic range that will meet the viral challenge of the season [55]. However, several problems in such manufacture remain poorly resolved. First, a stable supply of eggs is needed, which has required drastic reorganization of hen flock management [54]. Second, the egg-based viral culture is not efficient in producing high titers of virus with antigenic and genetic fidelity. Hence, there is a question of whether this procedure permits production on a scale that would be able to protect against a serious pandemic. Third, the potential susceptibility of chickens to HPAI viruses further jeopardizes the availability of eggs for vaccine production in the event of a pandemic strain arising from an HPAI virus [56].

A simpler approach to make influenza antigens in cell cultures routinely involves the synthesis of protein subcomponents of the virus using recombinant methodologies [57]. Several experimental enquiries are further exploring these possibilities by testing multiple host cell types for efficacy in producing different recombinant influenza antigens. HA was made in insect cells [58], the M_2_ protein in plant cells [59] as well as *Escherichia coli*, where this protein was conjugated to the TLR-5 ligand flagellin [60], or the heat shock protein (HSP)-70 of *Mycobacterium tuberculosis*[61]. As discussed further in “Innate immunity and immunogenic and physicochemical properties of adjuvants” section, these adjuvants are necessary since the subunit antigens are generally poorly immunogenic.

### In vivo-expressed influenza DNA vaccines

DNA vaccines are basically expression constructs that will produce the encoded antigen following delivery of the construct to an appropriate host cell. Compared with the production of whole viruses or viral subcomponents, DNA vaccines are easy to manufacture and extraordinarily stable when stored in the lyophilized state.

DNA vaccines have been applied as model systems to study the possibility of inducing antibodies against the HA, NP, and M1 proteins [62, 63], the ability to raise CTL response, and to investigate the protection in mice, chicken, ferrets and primates using intranasal instillation, intravenous injection, intramuscular injection, or gene gun [62, 64]. These experiments confirmed that influenza DNA vaccines can induce humoral and cellular responses, and the animal will get full or partial immunity to the challenge [62]. In mice, the antibody formation against HA was mostly of IgG2a isotype, but switched to IgG1 when the gene gun was used. IgG and IgA antibodies to HA were also secreted to the upper respiratory tract of vaccinated animals [65]. In primates, the titer was as high as the titer induced by human commercial influenza vaccines [66]. In mice, NP-coding DNA vaccines were able to increase the level of CD8+ CTL as well as the cellular immune response, which can give proper immunity to a wide spectrum of influenza subtypes [64]. Furthermore, vaccination with the influenza DNA vaccine coding NP helped to induce high levels of secretion of CD4+ Th1, and to increase the levels of interferon-γ and interleukin-20 [67]. Both CD8+ CTL and CD4+ T-cells gave protection against influenza challenge [65]. In view of the recent findings that interferon-γ-secreting CD4+ Th cells are important in obtaining protection against influenza virus infection [38], DNA vaccination may carry particularly beneficial properties in shaping the right phenotypic composition of influenza virus antigen-reactive T lymphocytes. However, in spite of nearly two decades of research, influenza DNA vaccination in clinical trials has not been an unqualified success, suggesting that both choices of antigens and adjuvants as well as the means of DNA cell delivery may need reconsideration [55].

The Flumist vaccine (Table 1) with live-attenuated virus represents an important alternative to obtain in vivo expression of influenza virus antigens, this way supporting T cellular responses. In this case, an attenuated master donor virus with appropriate characteristics with regard to cold-adaptation and stability is mixed in cell cultures with a potentially epidemic wild-type virus. This procedure reassorts the RNA segments of the master donor such that it will encode the HA and NA of the wild-type virus [68].

## Innate immunity and immunogenic and physicochemical properties of adjuvants

Vaccines are occasionally formulated with adjuvants to augment the potency of the antigen and presentation to the immune system. These co-administered adjuvants may enhance humoral and cellular immune responses simultaneously [69]. Adjuvants comprise a surprisingly diverse range of compounds, including mineral salts, oil-in-water emulsions, saponin-based adjuvants, liposomes, micropar-ticles, cytokines, and polysaccharides [70]. Pandemic influenza vaccines are formulated with adjuvant since they are typically monovalent and meant to protect immunologically naïve vaccinees. Among seasonal influenza vaccination, as noted from Table 1, none of the 2013–2014 seasonal vaccinations contains adjuvants. Nevertheless, as presented further below, several experimental approaches clearly support that improvement of adjuvant efficacy may be an important route to obtaining better protecting influenza vaccines or reduce the dosage needed to obtain sufficient protection.

Historically, vaccine adjuvants were developed empirically, initially based on an assumption that the antigen adsorption onto solids would prolong the stimulus *in vivo*[71, 72]. In a broad outline, their immunostimulating contributions are currently considered as relating to (1) chemical stabilization of vaccine antigens, (2) improving antigen delivery to antigen-presenting cells, (3) improving antigen processing and presentation by antigen-presenting cells, (4) stimulating the production of desirable immunomodulatory cytokines, and (5) permitting a decrease in the required dosage of the administered antigen. It is clear that the innate immune system is involved in all of these effects. In an important treatise defining the concept of innate immunity, Janeway noted that immune responses raised to soluble proteins in experimental models almost always required the further addition of “messy precipitates” [1], which apparently deliver a stimulus required for efficient formation of antigen-specific immunity. As noted in “Protein-based vaccines against influenza” section, indeed adjuvants are obligatory in vaccines based on viral subcomponents such as the pandemic influenza vaccines. Janeway concluded that this stimulus was triggered by “nonclonal recognition of nonself patterns” [1]. This is now widely recognized in the concept of adjuvants mimicking pathogen-associated molecular patterns [73, 74], and supported by observations on pattern-recognition receptor agonists used as adjuvants [75]. However, the particulate nature of many adjuvants has received less attention as part of the explanation for the mechanisms of adjuvants. As explored further below, the particulate nature of many adjuvants may add an ultrastructural feature to the vaccine formulation, which is likely to activate both humoral and cellular factors of the innate immune system.

### Licensed adjuvants in clinical use

Among adjuvants that have obtained a license in the European Union (Table 2), aluminum salts, oil-in-water emulsions (*e.g.*, MF59), alum-adsorbed TLR4 agonists (*e.g.*, adjuvant system [AS] 04), and liposomes (*e.g.*, Crucell), are the most widely used [80]. The list of licensed adjuvants in the United States is even more restricted, and includes only aluminum salts and AS04 [81] (Table 2).

**Table 2:** **Approximate diameters and chemical constituents of particulate adjuvants used in humans for prophylactic vaccination against viral infections** ^**a**^

***Adjuvant name (Provider; year licensed)***	***Particle size (nm)***	***Adjuvant type***	***Chemical constituents***	***Vaccines (virus)***
*Aluminum salt* (Various; 1924)	1,000-20,000^b^	Mineral salts	Aluminium hydroxyphosphate *or* Aluminum hydroxysulfate *or* Aluminum oxyhydroxide	Various
*MF59* (Novartis; 1997)	160^c^	Oil-in-water emulsion	Squalene, polysorbate 80, sorbitan trioleate	Fluad (seasonal influenza), Focetria (pandemic influenza), Aflunov (pre-pandemic influenza)
*AS03* (GlaxoSmithKline; 2005)	< 200^d^	Oil-in-water emulsion	Squalene; polysorbate 80, α-tocopherol	Pandemrix (pandemic influenza), Prepandrix (pre-pandemic influenza)
*Virosomes* (Berna Biotech; 2000)	100-200^e^	Liposomes	Influenza virus (lipid) envelope	Inflexal (seasonal influenza), Epaxal (hepatitis A)
*AS04* (GlaxoSmithKline; 2005)	1,000-20,000^b^	Alum-adsorbed TLR4 agonist	Aluminum oxyhydroxide, MPL	Fendrix (hepatitis B), Cervarix (human papilloma virus)

^a^Data reported are for the vaccine formulations with antigens. ^b^Values are from a review by Hem & HogenEsch [76]. ^c^Value from a review by O’Hagan *et al.*[77]. ^d^Value from a review by Garcon *et al.*[78]. ^e^Value from de Jonge *et al.*[79].

Aluminum salts are widely used as adjuvants and are included in the hepatitis B virus, papillomavirus, and diphtheria-tetanus-pertussis vaccines [72]. The majority of aluminum salt adjuvants used in humans comprise amorphous aluminum hydroxyphosphate (Al(OH)_x_ (PO4)_y_), amorphous aluminum hydroxysulfate (generated from precipitation of the antigen with AlK(SO_4_)_2_ named alum), and aluminum hydroxide, which is more correctly described as aluminum oxyhydroxide, AlO(OH) [76]. These salts are insoluble in water and form particles disperse in size (Table 2).

Studies have demonstrated the apparent superiority of aluminum hydroxide-adsorbed vaccines when compared to soluble adjuvants [82]. However, aluminum salts are ineffective in providing immunity against pathogens requiring Th1 mediated immunity [83, 84]. Their mode-of-action were previously considered to support antigen persistence *in vivo* by prolonging antigen release. This effect is often referred to as the “depot effect”, although its relevance has been questioned in recent reports [85]. The aluminum salt particles may rather serve to activate macrophages and dendritic cells [72, 86]. With regard to safety, aluminum salts have been commercially available for several decades [87], and are generally considered well-tolerated [88]. However, experimental evidence suggests that complexing of Al^3+^ ions with glucose-6-phosphate may interfere with the energy metabolism in a way that at least on speculative grounds might link high concentrations of aluminum to neural disorders or inflammatory syndromes [89].

Oil-in-water emulsions based on the natural lipid product squalene, *i.e.*, MF59, are licensed in most parts of Europe to be used with an updated seasonal influenza vaccine, primarily in elderly vaccinees. Moreover, MF59 and AS03 are the adjuvants of choice in pandemic influenza vaccines [74, 90]. Although made from a softer material than the aluminum salts, the squalene and polysorbate mixture nevertheless in aqueous environment manage to form stable droplets as a consequence of the hydrophobic effect [77, 78] and a diameter of ~100 nm [91]. As is also the case for mineral particles, the oil-in-water interface may adsorb protein from the medium, such as albumin [92].

In terms of the pharmacological mode-of-action, MF59 presumably acts by inducing a local immunostimulatory environment at the site of injection. This is characterized by enhanced antigen persistence, an increased antigen uptake by dendritic cells, and the recruitment of APCs [90, 93]. Oil-in-water emulsions induce stronger antibody responses, which reduce the need for multiple doses, and lead to a combined Th1 and Th2 memory response [94]. The administration of squalene has been associated with the development of arthritis in rats [95]. Nevertheless, the evidence so far presented does not suggest significant or frequent side effects prompted by the use of squalene adjuvant in humans [96]. The H1N1 influenza vaccination Pandemrix contains squalene. Application of the vaccination was suggested to be associated with the development of narcolepsy in children [97, 98], a disorder which involves the immune system [99]. However, it is unclear what role, if any, the AS03 adjuvant played in these clinical findings.

Other vaccines utilize a new class of adjuvant systems (AS04), which combine aluminum hydroxide and a proprietary form of detoxified monophosphoryl lipid A (MPL). MPL is derived from the gram-negative bacterium *Salmonella minnesota* R595 strain, and is a specific agonist of TLR4, comparable to lipopolysaccharide [100–104]. Indeed, lipopolysaccharide may also potentiate the immune response. However, frequent pyrogenic activities preclude the use of lipopolyssacharide as adjuvant for human use [72, 74, 83]. In contrast, the less toxic MPL is administered in vaccines for human use without any reported adverse effects [105]. AS04 directs a polarized Th1-response, as opposed to the Th2 response of aluminum salt alone. While the adjuvant activity of this formulation can mostly be ascribed to MPL, the “depot effect” of aluminum was suggested to prolong the stimulation by MPL [74, 83].

Similar to the more familiar liposomes, virosomes are composed of a phospholipid bilayer, but unlike liposomes, the bilayer is unilamellar and modified with viral envelope proteins (*e.g.*, in the case of influenza-like particles with NA and HA anchored in their virosomal membrane). In this way, virosomes are designed to bind and fuse with host cells similar to their cognate viruses. Antigens anchored to the surface are degraded upon endosomal fusion within the endosome and are consequently displayed to the immune system by MHC class II receptors. In contrast, antigens encapsulated within the virosomes are transported to the cytosol during the fusion event, which enables them to enter the MHC class I pathway of antigen presentation. Hence, virosomes possess the capability of mediating humoral as well as cell-mediated immune responses [106, 107]. Currently, two commercial vaccines utilizing the virosome technology have acquired a license: Epaxal® against hepatitis A and Inflexal® against influenza. Both are proven to be effective immunogens with unique adjuvant properties [108].

### New generation of adjuvants for influenza vaccines

Novel vaccine adjuvants are designed to favour stronger responses as well as the development of Th1, Th2, or CTL-mediated immunity. Cationic liposomes are such strong activators of the immune system. As already mentioned above, selective cellular responses may be required to offer efficient immunity to influenza [109]. To achieve this goal, genetic adjuvants and cytokines may be efficient. In general, genetic adjuvants are constituted by plasmid vectors encoding immunomodulatory products, such as cytokines [110]. Interestingly, novel directions in the design of genetic adjuvants include the use of DNA motifs such as CpG or HSPs from *Mycobacterium*.

Cationic liposomes are potent stimulators of the immune system [111, 112]. While there is a considerable literature on experimental use of such liposomes as adjuvant, it is only recently that promising clinical trials have been conducted [113]. At least two influenza vaccines with cationic liposomes as adjuvants have been tested in clinical trials [114, 115]; one addressing the complications mentioned in “Viral infectivity, treatment, and immunity to influenza” section of raising protective immunity in the elderly vaccinees [115]. The cationic adjuvant formulation no. 1 (CAF01) consists of dimethyldioctadecylammonium and α,α‘-trehalose-6,6′-dibehenate (TDB) [116]. TDB is a synthetic analogue of trehalose 6,6′-dimycolate, which itself possess an unwanted toxicity. However, by preserving the *Mycobacterium*-like lipid structure in TDB, the CAF01 formulation makes it possible to raise an immune response to antigens from *Mycobacterium*, such as the early-secreted antigenic target 6 kDa (ESAT-6). The mode-of-action of the adjuvant seems to involve the TLR-independent Syk/Card9-dependent pathway [116], apparently through direct binding to the C-type lectin receptor Mincle expressed in macrophages [117]. These findings points to an interesting principle in the choice of appropriate adjuvants also explored below, namely efficient adjuvants as a source of an innate immune response similar to those induced by the target microbial organism. As a tool to direct T cellular response the CAF01 cationic lipid composition appears to produce a Th1 response [118], but examples of cationic lipids stimulating Th2 response are also reported [118, 119].

Unmethylated bacterial CpG motifs are commonly used recombinant adjuvants that can induce innate immune response to DNA vaccines. Because CpG motifs in vertebrates are often methylated, bacterial CpG motifs are recognized as pathogen-associated molecular patterns by the human immune system, typically by TLR-9 after receptor-mediated endocytosis [120–122]. CpG motifs enhance both humoral and cellular immune responses to the encoded vaccine antigen, skewing the cellular response towards Th1 phenotype [123]. Recently, CpG DNA formed an important part in DNA vaccine protection against *Mycobacterium* tuberculosis, with ability of the adjuvant to augment the lung infiltrate of interferon-γ producing T lymphocytes [124]. This finding is not, however, independent of the microbial challenge or applied vaccine antigen. Wu *et al.* showed that addition of CpG DNA may even weaken the protective activity of the influenza M_2_ protein vaccine [125].

In three independent studies, *Mycobacterium*-dependent protein-1, HSP 70, and ESAT-6, respectively, were used as potential genetic adjuvants for the avian influenza H5 DNA vaccine [61, 126, 127]. HSPs are chaperones and play an important role in protein folding and prevention of protein aggregation or misfolding. Previous studies have shown the high potential of HSPs as genetic adjuvants in DNA-based vaccination, probably due to their capacity to stabilize weak antigens, permitting the delivery to APCs, notably DCs [128]. When fused to vaccine antigens, HSPs or heat shock cognate proteins can elicit CD8+ CTL response *in vivo* and *in vitro*[129]. Optimal results are obtained if they are directly fused with the vaccine gene of interest [61, 130, 131]. Rasoli *et al.* tested a potential enhancement of immunogenicity of an avian influenza virus DNA vaccine, where the H5 gene was fused with the HSP-70 gene [61].

### Adjuvants as particulate material and immunogenicity

Many commonly applied adjuvants precipitate in aqueous environment and remain stable as particles once administered suggesting this property to be important. With doubts being cast on the “depot” effect as rationalizing the effect of adjuvant [85], it remains enigmatic precisely why “messy precipitates” as adjuvants are such a provocation of the immune system. As detailed in an excellent review by Fox *et al.*[132], the physicochemical properties of particulate materials affect, however, many aspects of vaccine efficacy. Indeed, as noted above, other physicochemical properties such as surface charge of the particulate material has received interest, while the size of these particles are not often mentioned even though it is an intrinsic property of precipitates.

We propose that the colloidal nature of several adjuvants (Table 2) influence the immune system through three highly interconnected routes.

**Figure 2:**
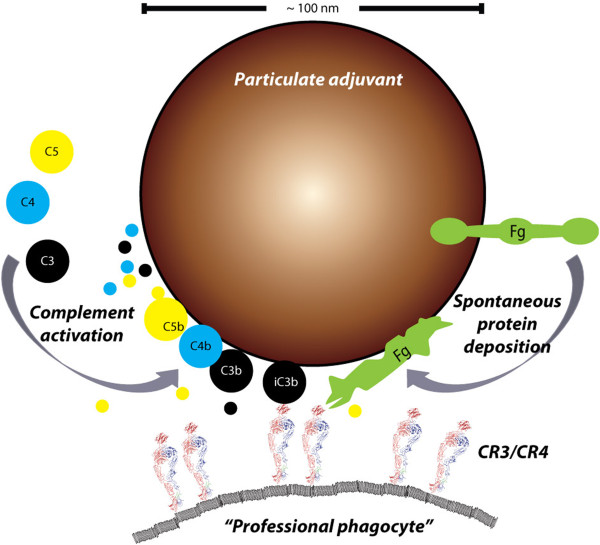
**The sources and consequences of plasma protein deposition on particulate adjuvants.** Deposition of complement is well-described for liposomes and is likely to happen on the surface lipid adjuvants as well [142]. The deposition of complement on aluminum oxide particles is less characterized [148]. As clear from the schematic, a particle with a diameter of ~100 nm presents certain space and topological restraints, which may influence both the complement activation and actually capacity for carrying deposited protein as detailed elsewhere [134]. Following activation of the complement components C3, C4, and C5 these are covalently bound to target surfaces concomitantly with the proteolytic release of small peptides from the molecules (indicated with colors). This release depends on the pathway of complement activation and may hence create a signature for adjuvants. In this scenario, which does not explicitely involve the vaccine antigen, the adjuvants act as by-standers creating a milieu of immunostimulatory peptides. Proteolytic processing of the C3b fragment creates the iC3b fragments, which is ligand for both CR3 and CR4 and may thus support phagocytosis by “professional phagocytes” [161] through the connection of these receptors with cytoskeleton. In addition to complement, surface-adsorbed fibrinogen is a ligand for CR3 and CR4, probably in part because of the structural denaturation of the protein as a consequence of the surface adsorption, which enhances the interaction with these receptors [155, 156]. Sizes are indicated approximately to scale based on data presented in Refs. [134, 166, 167].

First, the deposition of soluble proteins of the innate immune system on particle surfaces stimulates in many cases a proinflammatory response. Notably, these proteins include components of the complement system, which may amplify interactions with receptors on cells of the innate immune system. In this situation, peptides released by the complement activation make the adjuvants proinflammatory “by-standers”, which influence the vaccine response indirectly by activating leukocytes. Second, both the deposition of complement as well as a spontaneous deposition of other proteins on the adjuvant particle surface, such as the abundant plasma proteins fibrinogen or albumin may generate cues for recognition by receptors expressed on leukocytes. This is a less indirect route of stimulating leukocytes to vaccine antigen responses since such protein-coated adjuvants directly interact with the leukocytes involved in the process. Third, the biophysical characteristics of particulate material, *e.g.*, size and surface charge, may dramatically influence cellular uptake and response [133]. This phenomenon relates to both the rigidity of the cell membrane, and the accompanying molecular interactions permitting receptor recognition of ligands on the particulate surfaces Figure 2. As reviewed below, here it appears that size *per se* is an important factor in the direct interactions between the adjuvant and leukocytes.

The complement system is constituted by approximately 40 soluble plasma proteins and receptors. In essence, the function of complement involves the capability of the soluble proteins to transform into a surface-bound state with deposition on target surfaces, *e.g.*; the cell walls of bacterial or fungal organisms, envelopes or capsids of viruses, or even decayed host tissue or apoptotic cells. The pathways of activation have been detailed elsewhere [134]. Briefly, three distinct mechanisms initiate the deposition of complement. The classical pathway *(i)* is activated by binding of the complement component (C) C1 complex to IgG or IgM antibodies. Followed by proteolytic activation of C4 and C2, which generate the surface-bound fragments C4b and C2a and the small soluble fragments C4a and C2b, an enzyme C4bC2a is formed, which converts C3 into surface-bound C3b and the soluble fragment C3a. A similar process, named the lectin pathway *(ii)*, may be initiated by binding of MBL to suitable carbohydrates or by binding of ficolins to certain acetylated compounds [135]. Finally, the alternative pathway *(iii)* is based on the ability of C3 to deposit spontaneously on nearly all biological surfaces. Host cells may catalyze the removal of C3 and other complement components from their cell surface. By contrast, many microbial cells do not harbour such mechanisms, and are hence susceptible to complement attack. Following the covalent deposition of C3b, a complex with the proteolytic part of activated Factor B (termed Bb) is formed. Unlike C4bC2a enzyme, the C3bBb enzyme permits a positive amplification loop since the deposition of more C3b creates more enzymes. Further proteolytic degradation *in situ* generates the iC3b and C3d fragments, which are ligand for complement receptors (CR)2 and CR3. Also, the C4bC2a and C3bBb enzymes enables the recruitment of the large C5b to the surface, while releasing the small C5a peptide to the environment. C5a is a strong activator of the endothelium of blood vessels, and central in the recruitment of many inflammatory cells to the site of complement activation.

Complement has long been known to play a quintessential role of in raising a strong antibody response to antigens [136], now more fully understood with the help of intravital fluorescent microscopy of the cellular processes involved in the lymph node [137, 138]. A part of the benefit from complement activation may derive from the covalent coupling of a proteolytic fragment of C3 (C3d) to antigens, this way supporting the uptake by B cells through CR2. This has already been explored as a so-called “molecular adjuvant” in experimental studies by engineered coupling of this fragment onto target antigens, including influenza vaccine antigens [139–141].

A less appreciated route of complement influencing the function of adjuvants involves direct activation of complement. Indeed, it is well established that lipid vesicles in the kind of liposomes may activate the complement system [142, 143]. The *in vivo* significance of this phenomenon is well-known in the field of drug delivery. Such activation accelerates the clearance of the liposome from free circulation through capture by hepatic macrophages in a process, which is very similar to the fate of viral particles opsonised by complement. More recently, it has become clear that poloxamers, a type of pluronic block co-polymers, also activate the complement system as well as more widely affects cellular functions when administered [144, 145]. Poloxamers have been tested in the context of experimental DNA vaccines to influenza virus [146]. The benefit from this addition was probably in part obtained by improving DNA drug delivery [146]. Nevertheless, it is tempting to suggest that complement activation by poloxamers also contributed to the immunogenicity due to the complement system’s many influences on the inflammatory response. Interestingly, these poloxamers as well as drug delivery liposomes and lipidic adjuvants used in the clinic (Table 2) attain diameters of 100–200 nm [77–79, 146], similar to the dimensions of influenza virus particles with a diameter of ~120 nm (Figure 1) [19]. This is a conspicuous property of both the adjuvant and viral particles since Pedersen *et al.*[147] reported that nanoparticles with structural features similar to molecular initiators of complement activation produced stronger responses than particles with either smaller or larger sizes than these initiators. At least in serum from some donors the strongest activation was clearly found for dextran-coated polystyrene or iron oxide particles with diameters of 100–250 nm, apparently pending a sufficient titer of anti-dextran IgM. This effect was attributed to perturbations in the ultrastructure of IgM with cross-sectional diameters in the order of 20–30 nm [134], when IgM bound epitopes on the curved surface of the particles. With regard to aluminium salts and phosphate adjuvants, these are composed of small (~50 nm) primary particles, similar in size to the lipid adjuvants. The antigen-precipitated particles are, however, far larger than those generated from lipid adjuvants, typically with diameters ~1,000-20,000 nm [76]. This would seem to preclude complement activation through the processes addressed by Pedersen et *al.*[147]. Furthermore, while aluminium salt particles activate complement [148], there is evidence that at least hydrated aluminium surfaces adsorbed complement in a manner not generating the typical proteolytic cleavage products associated with activation [149]. This could point to significant differences between particulate adjuvants in their interaction with the complement proteins and hence in their immunomodulatory capacity and capabilities.

Obviously, the topology of particles is only one source of properties that may tune the ability to activate complement. By altering the interligand distance from 6 nm up 14 nm, an almost 1,000-fold increase in the dissociation constant was observed for the binding of the MBL oligomers to these surfaces [22]. This disproportional response, with a two-fold alteration in ligand density causing a 1,000-fold lowering of the affinity, was interpreted to reflect the critical difference between binding patterns of dimensions comparable to the size of MBL and those even just moderately exceeding these dimensions. As recently demonstrated by Pacheco *et al.*[150] for complement activation through particle surface-bound IgG, there is a complex interplay between size of the particles and amount of bound ligand for C1, *i.e.*, the IgG Fc parts, in regulating the strength of the activation [150]. Similar to the findings by Pedersen *et al.*[147], particles with a diameter of 500 nm appeared more efficient in activating complement than larger particles with a diameter of 4,000 nm. From careful quantification of the surface-bound IgG and multiple experiments varying the amount of IgG, Pacheco *et al.* hypothesized that an essential trigger of complement activation is assembly of the C1 complex via binding to multiple, closely apposed Fc parts [150]. C1 and MBL share several biochemical properties, in particular with regard to their ultrastructure and ability of both proteins to form polyvalent interactions. With this in mind, the critical role of polyvalency for strong MBL bonding to ligand-presenting surfaces [22], supports Pacheco *et al.* hypothesis [150].

As already noted in “Viral infectivity, treatment, and immunity to influenza” section, complement activation is an important part of immunity to influenza virus. The ability of adjuvant to trigger such a response is consequently mimicking conditions that could well be essential to enable full priming of the adaptive immune system, which is required to obtain antigen recognition. Importantly, in this scenario, the influence of complement activation is mediated by the adjuvant particles acting as “by-standers” creating a milieu of inflammation-stimulating complement peptides. More specifically, the generation of the smaller peptide fragments from C3, C4 and C5 may arguably create signatures of the type of complement activation induced. It may be significant that the adjuvant mimic the complement activation signature of the natural infection, which vaccination sought to prevent. For instance, liposomes appear to trigger a response through the alternative pathway [151] implying that primarily C3a and C5a are generated in this process. By contrast, as mentioned above, influenza virus is most likely activating complement through all pathways [152] with a prominent role of the lectin pathway, thereby suggesting the additional presence of C4a and C2b fragments. Yet, the influences of these peptidic signatures are only incompletely understood. As a valuable comparison on how small differences in proteolytic activities may change the profile of cleavage products, a recent report demonstrated vast alterations in the cleavage of soluble protein, and their cutaneous deposition during inflammation. This emerged as a consequence of genetic ablation in mice of a single protease, namely MMP-2 [153]. By analogy, we propose that regulating the adjuvant-triggered pathway of complement activation, and hence the proteolytic activities, potentially could quantitatively and qualitatively improve the outcome of vaccination.

An even simpler route to immune activation through the interaction between adjuvant surfaces and body fluid proteins is derived from the spontaneous surface deposition of notably albumin and fibrinogen [154]. Such spontaneous deposition on particle surfaces has been observed to affect or even destroy the structure of the adsorbed protein in particle-size-dependent manner [155]. Denatured fibrinogen is a ligand for CR3 and CR4 [156]. Hence, the surfaces of particulate adjuvants play an important role in permitting receptor-mediated contacts between adjuvants and leukocytes, probably in some cases by manufacturing a high density of cues for receptor binding on the particle surface. Such interactions are extremely strong [34] and may cause receptor signalling through CR3 leading to stimulation of cytokine production [157]. Obviously, the processes discussed above are initiated by simple contact of the colloidal adjuvant with protein-containing fluids, notably blood or interstitial fluids, which seems unavoidable with the chosen routes of vaccine administration. As outlined above and also noted from other studies [158], the transition of soluble proteins onto surfaces offers a wealth of complex quantitative influences, which may well manipulate those microenvironments-developing immune responses. Even so, it is an interesting observation that both past and present investigations into the adjuvant properties have largely ignored these processes and focused on the later cellular responses, such as modulated receptor expression and activation of the inflammasome. As noted by Fox *et al.*[132] this does not capture the full picture of the processes enabling the adjuvants to make such stimulations.

It is a classic observation that receptor-mediated endocytosis both have upper and lower limits with regard to the size range of particulate material that may enter the cell through a given mechanism [159, 160]. Pinocytosis and receptor-mediated endocytosis brings in to the cytoplasm relatively small entities such as macromolecules or viral particles, apparently through a shared use of clathrin [159]. Receptor-mediated endocytosis is most efficient with particles taking *radii* of 25–30 nm with a sharp cut in efficiency for *radii* smaller than ~22 nm [160]. These smaller particles may still enter the cells through pinocytosis albeit less efficiently. By contrast, phagocytosis enables uptake of particles larger than 500 nm in a process involving reorganization of the actin cytoskeleton. This process is typically mediated by Fc receptors, or the integrins CR3 and CR4 expressed on “professional phagocytes” [161], which includes macrophages/dendritic cells, monocytes, and granulocytes [159, 161]. On top of the size-selectivity in terms of the actual cellular process mediating the uptake, it is now clear that both micro- and nanoparticulate material influence these processes through their shape [162, 163]. This influence appears to derive from the ability of the cell membrane to form a phagocytic cup covering the particle [164].

In the context of adjuvants, it is of considerable interest that human B cells are unable to phagocytose particulates but may endocytose smaller particles [165]. This makes the large aluminium hydroxide particles unlikely to directly enter the cytosol of B cells, while the C3d-tagged antigens would easily do so. By contrast, the aluminum hydroxide particles can be phagocytozed by “professional phagocytes”, which during the intracellular storage may release the antigen from aluminium hydroxide matrix and permitting presentation of antigen by myeloid cells in the lymph nodes to CD4+ T cells. In this way, simple differences in size of the particulate adjuvant and modification of molecular adjuvant tags on the antigen could potentially qualitatively and quantitatively change the subsets of leukocytes involved in the formation of an immune response. While the size-difference between macromolecules and aluminium hydroxide particles are striking and simple to relate to the known capacity of certain types of cellular endocytosis, the lipid adjuvants with sizes intermediate between these extremes are far more difficult to assign similar discrete mechanisms. Indeed, based on theoretical calculations and the well-known ability of macrophages to clear liposomes [142, 160], their size regimen (Table 2) seems to indicate that both B cells and myeloid cells would be able endocytose or phagocytose these types particulates. At least on speculative grounds, this would suggest that minor differences in the size of these adjuvants could tilt the immune response towards either endocytosis or phagocytosis-mediated types of uptake hence altering immunological outcome of vaccination.

## Conclusions

Vaccination to protect against influenza infection is a field of great socio-economic and healthcare importance. The dual targeting of vaccine formulations, engaging both the innate and adaptive arms of the immune system, is crucial for efficacy. The TLR recognition of certain adjuvants is well documented and may hence serve as the prototype example for the claim that adjuvants are essentially enhancers of the innate immune response. The humoral part of the innate immune system, namely the complement system, may, however, well be equally important. In addressing the precise mechanisms of interactions between the adjuvants and the molecular environment of the body, we have stressed that in particular the size of adjuvants may fundamentally regulate their properties with regard to stimulating the immune response. The biology of influenza A viruses, notably their genetic flexibility, is significantly limiting the prospect of generating a single, omnipotent vaccine formulation. On the other hand, the conservative choice of adjuvants seems to ask the question if not more sophisticated strategies would now be of value, here perhaps in particular benefitting from the available scientific insight on mechanisms of the innate immune system. We propose that biophysical properties of the adjuvant formulation, in particular the dimensions of particulate materials, offer attractive opportunities to augment the immune response elicited.

## Abbreviations

APC: Antigen-presenting cell
AS: Adjuvant system
CAF01: Cationic adjuvant formulation no. 1
CR: Complement receptor
CTL: Cytotoxic T lymphocyte
DC: Dendritic cell
DNA: Deoxyribonucleic acid
ESAT-6: Early-secreted antigenic target 6 kDa
HA: Hemagglutinin
HPAI: High pathogenic avian influenza virus
HSP: Heat shock protein
Ig: Immunoglobulin
LPAI: Low pathogenic avian influenza virus
M: Matrix protein
MBL: Mannan-binding lectin
MPL: Monophosphoryl lipid A
NA: Neuroaminidase
NP: Nucleoprotein
RNA: Ribonucleic acid
TDB: α,α‘-trehalose 6,6*'*-dibehenate
Th: T helper cell
TLR: Toll-like receptor.
